# Review of “*Ecology of parasite-vector interactions*” by Willem Takken and Constantianus J.M. Koenraadt

**DOI:** 10.1186/1756-3305-6-72

**Published:** 2013-03-15

**Authors:** Andrei D Mihalca

**Affiliations:** 1 University of Agricultural Sciences and Veterinary Medicine Cluj-Napoca, Calea Mănăştur 3-5, Cluj-Napoca, 400372, Romania

## Book details

Takken D, Koenraadt CJM: *Ecology of parasite-vector interactions.* Wageningen Academic Publishers; 2013. 272 pages. ISBN 978–9086861880.

## Review

The increasing importance of vector-borne diseases is mirrored by the large number of papers published, by the emergence of dedicated new high-quality journals and also by a significant number of books published recently on this topic. This enormous quantity of data makes it difficult for scientists to filter the relevant high-quality from the average science. Excellent review collections, like this text, render the task more manageable. The third volume of the edited series “Ecology and control of vector-borne diseases” started in 2007, brings 12 contributed chapters grouped in three main sections: (1) Fundamental aspects of vector-parasite interactions; (2) Species-specific interactions and (3) Strategic issues concerning vector-parasite interactions. Despite the title of the book, which seems broad and general, the vast majority of contributions refer to mosquitoes and mosquito-borne diseases while other vectors are either poorly represented (i.e. ticks, sandflies) or completely absent (fleas, tsetse flies, triatomines etc.).

In the first section, the chapter “*Impact of transgenic immune deployment on mosquito fitness*” (by Pike *et al.*) presents brief reviews on the mosquito innate immune system and principles of mosquito transgenesis, followed by a detailed and well- referenced discussion on the impact of transgenic expression on the survival and fitness of mosquitoes under natural conditions. The chapter “*Plant-sugar feeding and vectorial capacity*” (by Stone and Foster) is an extensive and excellent review of the alternative feeding sources on plants of otherwise hematophagous insects followed by its impact on the vectorial capacity of mosquitoes and sandflies. “*Vector competence for arboviruses in relation to the larval environment of mosquitoes*” (by Alto and Lounibos) summarizes the results of studies on the effect of nutrition, competition, temperature and insecticides during the larval development on the vectorial transmission of arboviruses in adult mosquitoes. An overview on the risk prediction for malaria transmission depending on thermal variations is discussed in “*Relevant temperatures in mosquito and malaria biology*” (by Paaijmans and Thomas). A fine summary chapter, “*Evolutionary aspects of* Anopheles-Plasmodium *interactions*” (by Lambrechts and Koella) highlights the importance of the (co)evolutionary viewpoint for the better understanding of the history, distribution and dynamics of malaria transmission.

Included in the second section is an interesting and well-written article “*Tick-*Borrelia *interactions: burden or benefit*” (by Gassner and Hartemink). Based on experimental trials and field observations on the *Borrelia*-induced behavioural changes in ticks, the authors propose a theoretical model for assessing the impact of the fitness effect of the infection on larval survival. A brief, but very well- documented section is “Wolbachia *in* Aedes *mosquitoes: towards biological control of vector-borne diseases*” (by Moreira). The author reviews the potential value of using the endosymbiotic bacterium *Wolbachia* as a possible practical solution for the biological control of human vector-borne diseases. The chapter “*Behaviour of sandflies infected with* Leishmania” (by Ready and Rogers), reviews the mechanisms of manipulation by *Leishmania* of sandflies feeding on mammalian hosts in order to enhance parasites transmission, as well as the experimental models to study such pathogen-vector interactions.

In the third section, the chapter “*Modelling the control of mosquito-borne diseases*” (by North and Hancock) is a very well-focused overview on the strategies for controlling the mosquito vectors, with two broad issues considered: population dynamics and assessment of the impact of human interventions on population dynamics. Bousema and Baidjoe, in their review “*Heterogeneity of malaria transmission: underlying factors and implications for disease control*” discuss the factors influencing the existence of malaria transmission hotspots, and the practical use of these hotspots for targeted malaria control. Another interesting review, “*Considerations for male fitness in successful genetic vector control programs*” (by Helinski and Harrington) examines the male mosquito biology, which has otherwise received much less attention than their female counterparts. Furthermore, the authors emphasize the importance of transgenic and sterile male fitness studies under semi-wild conditions, rather than laboratory set-ups.

The editors of this book (Koenraadt and Takken) provide an excellent summary chapter emphasising how the understanding of fundamental aspects of the biology and ecology of parasite-vector interactions can be used in the practical sustainable control of vector-borne diseases.

Overall, this third volume of the series is very well edited and formatted, with an easy-to-read layout and very novel topics in the field of vector-borne diseases and vector-pathogen interactions. I am convinced that the target readership (parasitologists, entomologists, microbiologists, epidemiologists) will enjoy this book and will look forward for the next volume in this series.

## Competing interests

The author declares that he has no competing interests.

